# An Exploratory Investigation into the Roles of Critical Care Response Teams in End-of-Life Care

**DOI:** 10.1155/2021/4937241

**Published:** 2021-07-23

**Authors:** Adrienne Kwong, Stephanie Chenail, Aimee Sarti, Laura H. Thompson, Marlena Dang Nguyen, Kwadwo Kyeremanteng, Michael Hartwick

**Affiliations:** ^1^Division of Palliative Care, The Ottawa Hospital, Ottawa, Canada; ^2^Institut du Savoir Montfort, Ottawa, Canada; ^3^Division of Critical Care, The Ottawa Hospital, Ottawa, Canada; ^4^Ottawa Hospital Research Institute, Ottawa, Canada

## Abstract

**Background:**

Critical Care Response Teams (CCRTs) represent an important interface between end-of-life care (EOLC) and critical care medicine (CCM). The aim of this study was to explore the roles and interactions of CCRTs in the provision of EOLC from the perspective of CCRT members.

**Methods:**

Twelve registered nurses (RNs) and four respiratory therapists (RTs) took part in focus groups, and one-on-one interviews were conducted with six critical care physicians. Thematic coding using a modified constructivist grounded theory approach was used to identify emerging themes through an iterative process involving a four-member coding team.

**Results:**

Three main perspectives were identified that spoke to CCRT interactions and perceptions of EOLC encounters. CCRT members felt that they provide a unique skill set of multidisciplinary expertise in treating critically ill patients and evaluating the utility of intensive care treatments. However, despite feeling that they possessed the skills and resources to deliver quality EOLC, CCRT members were ambivalent with respect to whether EOLC was a part of their mandate. Challenges were also identified that impacted the ability of CCRTs to deliver quality EOLC.

**Conclusions:**

This research aids in understanding for the first time CCRT roles in EOLC from the perspectives of individual CCRT members themselves. While CCRTs provide unique multidisciplinary expertise to evaluate the utility of intensive care treatments, opportunities exist to support CCRTs in EOLC, such as dedicated EOLC training, protocols for advance care planning, documentation, and transitions to palliative care.

## 1. Introduction

Critical Care Response Teams (CCRT) [1] respond when urgent medical issues arise anywhere in the hospital, thereby extending crucial critical care expertise to all hospital units. The literature presents somewhat contradicting results regarding the impact of CCRTs on mortality and intensive care unit (ICU) admissions; however, there is general agreement that CCRT teams serve their intended purpose to improve patient outcomes by involving health providers trained in resuscitation more rapidly [1–8]. It has also been established that a proportion of CCRT calls involve helping to manage situations at the interface between critical care resuscitation and palliation/end-of-life care (EOLC), with EOLC issues being present in approximately 30% of CCRT interventions [9, 10]. A US-based study found that while CCRT inventions often resulted in ICU admissions, they also facilitated critical discussions regarding goals of care and frequently resulted in a transition toward palliative care strategies [11]. In contrast, another Ontario study found that there were fewer ICU admissions, shorter ICU length of stays, and an increase in palliative care consultations among patients whose end-of-life (EOL) status changed after a discussion with the CCRT [12]. A retrospective study investigating the impact of CCRT consultations in Ontario found opportunities to facilitate EOLC were often missed [13]. In addition to providing opportunities for EOLC, CCRT encounters also provide the opportunity to identify patients for whom a ‘not-for-resuscitation' (NFR) order should be considered [14, 15].

Although many studies have documented the participation of CCRT in EOLC, considerable gaps remain in understanding the nature of these encounters from the perspectives of the healthcare professionals involved. Specifically, it is unclear whether CCRT members perceive EOLC as a part of their mandate. If so, do they embrace this role? What additional skills and resources are perceived to be required to provide quality EOLC? And do team members feel they possess these skills? The aim of this study was to explore the roles and interactions of CCRTs in the provision of EOLC from the perspective of CCRT members.

## 2. Methods

### 2.1. Study Design and Participants

In order to better understand the perspectives of individual CCRT members regarding their roles and interactions in the provision of EOLC, a qualitative approach utilizing a modified constructivist grounded theory methodology was selected [16]. Members of the CCRT who participated in this study included 12 registered nurses (RNs), 4 respiratory therapists (RTs), and 6 critical care physicians (2 females, 4 males) at two academic tertiary care hospitals in Ottawa, Canada. Maximum variation sampling was used to recruit physicians with regard to location of practice and career stages, while RNs and RTs were selected based on availability and interest in participating. In our institutions, the CCRTs were established in 2005 and are referred to as ‘Rapid Assessment of Critical Events (RACE)' teams. For an RN to be a CCRT member, he or she must have at least 5 years of critical care experience. All CCRT calls are led by physicians and attended by both a dedicated critical care RN and RT. During daytime hours (8 : 00–17 : 00), the CCRT physician is a staff intensivist (MD). At night, the MD role is filled by a resident physician with a staff intensivist available for support. The remainder of the CCRT is unchanged at night. The study sites were sampled due to their different patient population in order to capture any nuances in local practices. Ethical approval for this study was granted by the Ottawa Health Science Network Research Ethics Board (REB). Informed consent was obtained from study participants prior to study participation.

### 2.2. Data Collection

Six, one-on-one, semistructured interviews averaging 40 minutes were conducted with physicians and six semistructured focus groups averaging one hour were conducted with RNs and RTs between March 2014 to May 2014. A multidisciplinary panel of experts in critical care, palliative care, and qualitative methodology reviewed and contributed to the development of interview guides for focus groups and interviews. Interviews and focus groups were audio recorded and transcribed verbatim.

### 2.3. Data Analysis

Interviews were analyzed by two researchers and individuals with CCRT experience including a physician and a RN. The interprofessional coding team participated in coding training and developing of a code book, with all team members agreeing on themes and codes. Coding was inductive with codes emerging directly from the datasets. Intercoder reliability was >85% concordant before independent coding was commenced. This occurred at approximately the midpoint of data coding (seven transcripts were coded with the team). The interviews and focus groups were all coded and entered into NVIVO software. Themes were generated directly from the dataset. Interviews and focus groups continued until saturation of themes was reached.

## 3. Results

The perspectives of twelve RNs, 4 RTs, and 6 critical care physicians, all members of the CCRT at two academic tertiary care hospitals in Ottawa, Canada, were included. Three main perspectives were identified that spoke to whether CCRT members perceive EOLC as part of their mandate, whether they embraced this role, and whether they felt like they had the skills and resources required to provide quality EOLC. Additionally, nighttime, system challenges, and lack of patient/family understanding of complex medical situations were three identified challenges that impacted the teams' ability to deliver quality EOLC.

### 3.1. Leveraging the Collective Wisdom from a Multidisciplinary Team, “Together We Can Have a Sense of the Whole—That Sense Is Extremely Important”

Members on the CCRT described how collective wisdom was valued, and expertise was derived from the experience of all team members. While all members on the team are individually competent, participants articulated how the unique ability to collaborate on decision-making gave the CCRT confidence when they encountered situations requiring EOLC discussions with patients and families and in giving recommendations to other members of a patient's circle of care (Table 1, Statement A).

While reaching a consensus was central to the CCRT decision-making, physicians on the team are still expected to make the final decision regarding plans of care, including decisions regarding EOLC. This expectation can be challenging when the staff physician is absent, and a less-experienced resident physician feels insecure with decision-making around EOLC (Table 1, Statement B). In situations where the CCRT RN or RT believed that a resident was uncomfortable with the situation at hand, the CCRT RN or RT described how they would often take on a leadership role and offer suggestions and/or guidance regarding patient care (Table 1, Statement C).

### 3.2. Knowing When ICU Treatment Is Needed, Knowing When EOLC Is Needed, “I Think There's Been a Lot More Dignity in Death than There Was before RACE Started”

A major perspective shared by all members on the CCRT was that they felt they possessed the skills and resources to provide quality EOLC when it was required. Several CCRT members articulated how their experience with treating critically ill patients in the intensive care unit allowed them to feel confident in their evaluations of complex cases to determine when a patient would benefit from intensive care management versus when supporting an EOL course was more appropriate (Table 1, Statement D). For example, CCRT members described how they are able to provide information regarding prognostication in critical illness, accurately describe the implications of initiating intensive care interventions, and clearly communicate plans for realistic, patient-centered goals of care (Table 1, Statement E). Furthermore, physicians on the CCRT felt that families were usually more receptive to communicating with them regarding EOLC, perhaps because families felt more reassured when the decision was coming from the critical care consultant (Table 1, Statement F). This ability to understand when a patient was at the end of their life enables the CCRT to recommend a plan of care that CCRT members perceive as more dignified (Table 1, Statement G).

### 3.3. Reluctance in Embracing EOLC as Part of Their Role, “Someone's Got to Do It”

Despite feeling that they possessed the skills and resources to deliver quality EOLC, many members on the CCRT did not feel that EOLC was part of their mandate, and members voiced reluctance in embracing this role.

While members on the CCRT felt that EOLC was an important facet of their role (Table 1, Statement H, PHYS03), many felt that they were often inappropriately left in a position to have EOLC discussions with patients with whom they have no rapport and who have chronic life-limiting illnesses. In these instances, the frustrations brought up by the CCRT centered around concerns regarding the relational and social aspects of patient care. They felt that most of these discussions should have occurred before an acute event and with a member of the most responsible service caring for the patient (Table 1, Statement H, RN).

An additional factor that contributed to reluctance in embracing EOLC as a part of the CCRT role was that they felt limited by the lack of in-depth familiarity with patients' cases, which could result in inaccurate prognostication. In these situations, it was widely acknowledged by many on the CCRT that their involvement needed to be balanced and checked by specialist expertise from the treating team (Table 1, Statement I). While the feelings of uncertainty expressed in this perspective seem to contradict with the prior perspective of having a certain level of expertise in predicting when a patient was at the end of their life, this tension highlights the need for collaboration with the treating team to ensure that the full picture of a patient's clinical status is well understood before decisions regarding EOL are made. Despite this, CCRT members still felt that these discussions were often deferred to them because of the treating teams' perceived discomfort or lack of expertise in EOLC (Table 1, Statement J).

Although CCRT members voiced ambivalence regarding being involved in EOLC discussions, they engage in these discussions because they felt that, otherwise, these important conversations would not take place. They ultimately felt that “somebody's got to do it” (Table 1, Statement K, PHYS01 and RT).

## 4. Challenges

The majority of CCRT members did not view EOLC as part of their mandate but engaged in it as it is necessary to support patients and their families because they had the expertise, comfort level, and experience in doing so. Nevertheless, they articulated the existence of several challenges impacting their ability to deliver quality EOLC.

### 4.1. Nighttime, “It Can Be a Lot More Stressful Being on at Night”

Members on the CCRT consistently described how CCRT function was impacted by the timing of CCRT calls. Ultimately, patient and family rapport, familiarity with a patient's clinical course, and specialist expertise are all aspects of patient care provided by a treating team that may not be present during nighttime hours. These clinical variations have the ability to significantly affect decision-making, particularly if an inexperienced resident is taking on the role of CCRT physician overnight and is not open to suggestions from the rest of the team (Table 1, Statement L).

### 4.2. System Challenges, “Patients Come in and Somebody Has Taken the Time to Have an End-of-Life Discussion with Them and You Cannot Find Them”

CCRT members also highlighted how technical system challenges impacted their ability to have quality EOLC discussions with families. At the time of this study, the ability to discover prior EOLC discussions and decisions in the medical record was limited, often only indicating the directive to offer or withhold resuscitative efforts (Table 1, Statement M).

### 4.3. Lack of Understanding Influencing EOLC Discussions, “Most People Become Receptive If You Have Someone Who Can Explain It Properly”

One of the most significant challenges to engaging in EOLC discussions emphasized by members of CCRT is related to dealing with patients and family members, who they perceived as not understanding the full implications of subjecting their critically ill family member to invasive treatments in the intensive care unit. Faced with these challenges, CCRT members spoke about how there was a need to very clearly communicate to family members the nature of the treatments, in particular the level of discomfort and invasiveness of the procedures involved (Table 1, Statement N). Some CCRT members felt that the degree to which a patient or family member understood a clinical situation was closely related to the practitioner's ability to communicate clearly (Table 1, Statement O). However, others felt that receiving “bad news” can impact patients' or family members' ability to absorb information even if clearly communicated by the practitioner (Table 1, Statement P).

## 5. Discussion

In this qualitative study conducted at two academic tertiary care hospitals in Ottawa, Canada, we interviewed CCRT members to better understand their experiences and interactions with EOLC. We found that members of the CCRT feel they possess the competence to treat critically ill patients and the expertise to accurately predict the utility of critical care for those approaching EOL. However, despite feeling that they possessed the skills to deliver quality EOLC, ambivalence was expressed as to whether CCRT members fully embraced EOLC as a part of their mandate. The principal finding is that CCRT members feel they do have the skills to provide EOLC. The ability to draw upon each team member's experience to act as an EOLC consultative resource was identified as an important strength of the multidisciplinary CCRT. Previous studies found EOLC to be a consistent component of CCRT consultation [9–15]. In particular, the integration of EOLC in CCRT consultations has been associated with a reduced number of ICU transfers, increased access to palliative care services, and improved advanced care planning [9–12, 14, 15]. Importantly, these studies highlight the relevance of certain characteristics and skills in the execution of EOLC within CCRT [9–15]. In addition to this, findings from our study suggest that distinct from dedicated training in EOLC skills, the composition of the CCRT is important. This mirrors the findings of Sprung et al. in the WELPICUS study which emphasized the importance of a multidisciplinary team in decision-making about EOLC and that this team should be comprised of “experienced intensive care specialists (intensivists), intensive care nurses, and other ICU professionals” [17].

In our study, we found that the inclusion of RNs and RTs with relevant critical care experience on the team was crucial to supporting CCRT resident physicians who may be inexperienced with EOL issues during the nighttime hours. Despite the strength of a multidisciplinary team, several recent studies in Canada and in the United States have highlighted differences in clinical outcomes during nighttime CCRT calls compared to daytime hours [18, 19]. These studies found that nighttime rapid response team (RRT) activation was associated with increased odds of mortality when compared with daytime RRT activation, attributing this higher risk to workforce shift changes, reduced patient/nursing ratios, and less-experienced resident physicians [18]. Our findings stress the importance of identifying strategies to support decision-making for CCRTs during nighttime hours.

Interestingly, we also found that although CCRT members felt they uniquely qualified to assist with EOL decision-making, they also believed that they were inappropriately placed in a position to do so because of the treating teams' discomfort or lack of expertise in having EOL conversations. This perspective has been found in other studies; in particular, a Canadian multicenter survey of medical teaching units found that the most significant deterrent to engaging in goals of care discussion among clinicians, hospital staff, residents, and nurses was fear around family members and patients' difficulty accepting poor prognosis [20]. Our findings reinforce the need for increased and improved training for multidisciplinary team members in EOL discussions [20–22]. Furthermore, our study found that CCRT members believed that interacting with patients with whom they had no rapport or familiarity limited their ability to deliver quality EOLC. Indeed, a review of best practices on communication about serious illness care goals found that conversations about goals of care were often conducted by physicians who did not know the patient well, resulting in a failure to provide patients with sufficient information about prognosis, ultimately leading to inappropriate decisions [21].

Downar et al. found that while RRTs frequently participated in EOL discussions and decision-making, consultations rarely led to palliative care, spiritual care consultations, or other comfort care measures [13]. This highlights the need for interprofessional collaboration with specialists in palliative care to improve EOLC interactions. While education in core EOLC skills has been broadly recommended for critical healthcare professionals [17], partnerships between CCRTs and consultative palliative care services to manage symptoms or assist in goals of care discussions should be encouraged. Nelson et al. proposed several strategies to improve the integration of palliative care and rapid response services [23]. These strategies include training CCRTs on core palliative care knowledge and skills and initiating an institutional effort to facilitate advance care planning and related documentation, in addition to identifying an interprofessional group of individuals who can support the CCRT in addressing palliative care needs of patients and families [23, 24].

Lastly, we also found that in instances where EOLC discussions and decisions had been made, there was no clear way to document and communicate to the CCRT what these decisions were. In a recent study auditing instances of advance care planning, it was found that nearly 30% of the patients and families who reported a preference for life-sustaining treatment did not have any documentation of such preferences in the medical record [25]. Furthermore, the majority (two-thirds) of preferences documented were discordant with patient's or family's expressed preferences [25]. This points to a significant issue and stresses the need to better coordinate patient care including implementing strategies to document all conversations regarding goal of care in a way that is easily accessible for others. Nelson et al. raised this important issue and have proposed strategies arguing for institutional changes to facilitate documentation regarding relevant advanced care planning (ACP) information [23].

Our study has a number of strengths. To our knowledge, it is the first qualitative study that seeks to understand the roles of interactions of CCRTs in the provision of EOLC from the perspective of CCRT members themselves [26]. The qualitative nature of our study resulted in findings conceived through iterative analysis made up of a multidisciplinary team of researchers. The results of this study provide important insights on how to support CCRT in providing EOLC including improving ACP procedures and interprofessional collaboration. Despite these contributions, our study has limitations. The perspectives obtained were only from CCRT members at two academic tertiary care hospitals. Data on the sex, age, and specific number of years of critical care experience for all team members were not collected. Furthermore, CCRT members experiences are situated in a CCRT configuration comprising a physician, a RN, and a RT. Centers that have, for example, a nurse led CCRT may have different perspectives with respect to managing EOLC situations.

## 6. Conclusions

The results from this study describe for the first time the perspectives of CCRT team members on their role in providing quality EOLC in two large Canadian university hospitals. CCRTs integrate experience from all team members and use this collective wisdom to act as a consultative resource to other members of a patient's circle of care. CCRTs provide a unique expertise in evaluating the utility of intensive care treatments; however, team members at times struggle to integrate their expertise into the advance care planning system of the hospital, leading to duplication, role confusion, and discontinuity. Since the data collection period for this study, no institutional changes to the CCRT base model or EOLC processes have been implemented. Our data highlight the potential for enhancement of overall hospital practice and protocols for advance care planning, documentation, and transitions to palliative medicine service, which would serve to complement the role of CCRT. Future studies could build on these findings by surveying members across various regions and specialties to determine differences in perspectives between providers and organizations.

## Figures and Tables

**Table 1:** Participant remarks.

Statement	Participant	Quotation
A	PHYS03	“...we have a tremendous degree of wisdom between us that's very helpful and I think that may be why we can make decisions [...] because we've got a group of people.”
B	PHYS02	“You're asking a resident to go out and see somebody who probably presumably doesn't have the same experience or knowledge as staff physician does, hasn't been in the scenario as often and so to start talking about end of life stuff, you have to be fairly confident that you understand what's going on with the patients and truly, you know, there's not something that you can do to make it all better. And you also have to be comfortable with talking about end of life stuff.”
C	RN	“Residents who are insecure about making decisions and we come and as a team we say, you know, this is not appropriate care. It's not appropriate to be putting this person on a ventilator and prolonging their death. It's not going to enhance their life in any way, and then we facilitate the resident getting to that point, maybe helping set up.”
D	PHYS06	“the people on the RACE team have a better understanding or appreciation, I guess, of when a patient looks like they're at end of life or in specific patients, do we think that they're actually... that we could actually fix them with invasive measure in the ICU and give them a reasonable outcome. I think we have a bit more or often a lot more baseline knowledge of that than initial providing services.”
E	PHYS04	“We've had these discussions many, many, many times. We also have seen so many patients that we know which, you know, who of these patients have a chance of surviving and who doesn't, [...] we can anticipate what's going to happen. And I think that plays a very important part of the discussion you can have with the family.”
F	PHYS04	“... families often feel more reassured when the actual provider of critical care says critical care is not appropriate in this situation. That it comes better from the word of mouth of the person who's actually offering or providing the treatment rather than the providing service.”
G	RN	“I have to say though, since the beginning of RACE, I think there's been a lot more dignity in death than there was before RACE started. I think we've avoided a lot of admissions to ICU because of RACE. I think we have done a lot of end of life at the bedside.”
H	PHYS03	“I think I felt very strongly that it was a very important facet of RACE but I often felt that I was not the appropriate person to be doing that and that... that we had been forced into a situation in which we're making the decision because the staff doctors had either not broached the situation adequately or had not appreciated the situation and the severity of the illness [...]”
	RN	“they called us into the room, and we have to have an end of life discussion with a family that we've never seen before. We have no rapport with them. It happens time and time again. And it's inappropriate in that regard, I mean, certainly when it's acute, or the patient's new, it's at least acceptable and we're better at it than a lot of people, but it seems like a lot of services don't accept that fact that people die.”

I	PHYS05	“I've gone to see a patient where I'm not sure that there's much that we can offer but then you speak to the attending service and find out that the baseline is quite a bit different than what your expectation was[...]. And we, ourselves, even when it comes to end of life care, we can't be so drawn into our own opinions that we're closed to other people's [...] I've changed my goals based on what other physicians who know patients better...the patient better than I do. So, I do worry about RACE and in that regard because we're the cavalry coming in and giving an opinion on the patient we don't necessarily know as well as the attending team.”
J	PHYS05	“I think some people are just uncomfortable telling it like it is and just being honest when they know that there are options that can temporize but not necessarily treat. And there's a big difference between survival and recovery for patients. [...] Sometimes I think the attending services are worried about consulting palliative care right off the bat so it almost seems like, “let's get RACE to see, see if there's anything we can do. If there's nothing we can do then the palliative care comes in [...] So where I think that there's lots of times when the answer is no, it's just they're afraid to say it or there's some barrier to that and having the discussions. [...] it's a comfort thing.”
K	PHYS01	“I think that somebody's got to do it and we have a good perspective to do it in terms of like expertise, the problem is that we don't have the relationships. So, from a relationship point of view, it'd be best to have the family doc or the primary treating service in the hospital. But usually those two groups don't have very much expertise in knowing who's a good candidate and who's not. They don't have the experience in having discussions either. [...]I've always felt that that was one of our roles because the alternative is much much worse. So we may not be the perfect person to do it but if we don't do it, nobody does it. Then you'll end up with a lot of people who shouldn't come to the ICU ending up in the ICU.”
	RT	“But do I think personally that we should get involved with all end of life discussions? Absolutely not. I agree with palliative care. Absolutely. That's their bread and butter even more than critical care. I think they should be involved more. but if we're already involved, we're involved. We're there. We're not going to neglect a patient. Never ever.”

L	PHYS05	“...sometimes we've had patients in the ICU who are there for three months. You discuss. You get to know them well. You write your opinion. It's all written down. And then a junior resident in the middle of the night gets called and changes goals of care with no understanding whatsoever of what that means.”
	RN	“It can be a lot more stressful being on at night. It's okay if you have a resident that's open to ideas. But when you have a resident that thinks they know it all and, you know, doesn't want to listen, you know, not open to things, doesn't want to call their senior to... that can be quite frustrating.”

M	PHYS02	“Other fascinating thing that's come up a few times is patients come in and somebody has taken the time to have an end of life discussion with them and you can't find them. So, it might be on the chart written in [a] progress note and the... you may stumble upon it, you may not stumble upon it. But in our Oacis system there should be a way of flagging a document that discussion has been had..”
N	RT	“I've often said this to residents, is, “You know, you can't just say, we're going to help them. Do you want us to help your family member?” Because everyone is going to say yes, we do. But if you say, you know, we're going to put them on life support, they may never come off life support. This is going to be a very uncomfortable tube for them to have put in their throats. They will not be able to talk to you or eat. When you start saying those words, then suddenly family, oh wait a minute, no they wouldn't want this. No, no, no.”
O	RN	“...families are more receptive to understanding that, you know, yeah everyone dies in the end and we could help make it a comfortable transition. Of course, you'll have your, you know, couple percent that still want everything but most people become receptive if you have someone that can explain it properly.”
P	PHYS02	“So as everyone learns in medicine, that you tell patients something that doesn't sound good, most of what they retain is very limited. And so even though we thought we had a conversation where we thought we expressed ourselves very well, it may not have been understood to the same degree. So, you know, the doctor and patient go separate directions with a different, completely different idea of what that conversation was just about. I think that happens an awful lot.”

## Data Availability

The qualitative data used to support the findings of this study are included within the article.
